# Supplementary material for: Toll-like Receptor 4, Osteoblasts and Leukemogenesis; the Lesson from Acute Myeloid Leukemia
Source: Molecules. 2022 Jan 23;27(3):735. doi: 10.3390/molecules27030735 (PMC8838156; doi:10.3390/molecules27030735)
Supplement: Supplementary file 1 [file molecules-27-00735-s001.zip › molecules-1480617-supplementary.pdf]

SUPPLEMENTARY INFORMATION

Toll-like Receptor 4, Osteoblasts and Leukemogenesis; the Lesson from Acute Myeloid Leukemia

Øystein Bruserud [1,2,*], Håkon Reikvam, [1,2] and Annette Katharina Brenner [1]

Table S1. TLR4 ligands and their possible expression in the bone marrow, effects on bone marrow stromal cells and possible relevance for regulation of hematopoiesis. The information in the table is based on the Gene database, the Human Protein Atlas (both accessed on September 6, 2021) and selected references in the PubMed database as indicated in the list after the table. [16,129–163]

LIGAND	FUNCTION
Tenacin	This is a glycoprotein associated with the extracellular matrix. It is expressed in human bone marrow; and tenascin C knock-out mice have defective hematopoiesis.
Hyaluronic acid	This extracellular matrix molecule supports normal hematopoiesis, the systemic serum levels are altered in AML.
Heparan sulfate	This proteoglycan is an extracellular matrix molecule; AML cells show increased expression and patients with the highest expression seem to have an adverse prognosis. It is important for normal hematopoiesis and the formation of the stem cell niches.
Fibronectin	Experimental studies suggest that AML cell adhesion to fibronectin increase leukemia cell proliferation, accelerate S-phase entry and cause accumulation of the cell cycle inhibitor CDC25A. This CDC25A accumulation was caused by decreased degradation. Activation of PI3K-Akt-mTOR seemed to be important for this adhesion-dependent growth enhancement. In contrast, fibronectin adhesion inhibited the proliferation of normal $CD34^+$ bone hematopoietic cells.
α1-microglobulin	This 183 amino acid glycoprotein is present in extracellular fluid and is a regulator of normal hematopoiesis. It is secreted by most cells. It has reductase activity that is non-specific with regard to substrate, and it can function as a scavenger protein.
α2-macroglobulin	The protein can be released by various mesenchymal cells, including osteoblasts It can be detected in extracellular fluid; it functions as a proteinase inhibitor and also as a carrier protein for several growth factors/cytokines, including several regulators of hematopoiesis and local angiogenesis. It has a protective effect on mesenchymal stem cells. It can bind and/or degrade several.
High mobility group protein B1 (HMGB1)	This chromatin-binding protein is also released extracellularly during necroptosis or by active secretion. Is expressed in AML cells, and then it has an antiapoptotic effect and functions as a regulator of proliferation, differentiation and autophagy. The extracellular form is regarded as a proinflammatory cytokine; it can bind several cytokines and chemokines that are involved in the regulation of both normal and leukemic hematopoiesis, both to TLR4 and RAGE glycoprotein receptor but possibly also to other receptors. Animal studies suggest that it is a regulator of normal hematopoiesis.
Vitmin D binding protein	Clinical studies suggest that the extracellular form of this protein is a modulator of cytokine secretion.
HSP60	This chaperone seems to be release to the extracellular space by exosomes and is probably important in cell-cell communication.
Fibrinogen	The systemic fibrinogen levels are increased in AML and high levels at the first time of diagnosis are associated with adverse outcome; this is not due to an effect on early mortality. Both soluble and solid-phase fibrinogen induces Syk signaling in human megakaryoblastic cell lines, and it can enhance IL3-dependent progenitor proliferation. Fibrinogen can also bind to integrins.
Amyloid-A	This is an extracellularly released acute phase protein that can bind both to TLR4 but also TLR2 and RAGE receptors. Both hepatic and extrahepatic release seems to contribute to the systemic levels.
Amyloid-β	The protein can be detected in the circulation and it can be released by bone marrow cells.
Lipids (oxidized LDL, fatty acids)	The TLR4 ligands include low density lipoproteins (LDL) and free fatty acids. Oxidized LDH modulates the differentiation of bone marrow MSCs, and various fatty acids can modulate AML cell proliferation.

Table S2. A summary of important TLR4 characteristics [PMID: 29867550, 30365988, 26136385, 27483231] [8, 17, 18, 19].

Endogenous TLR4 ligands [PMID 29867550]

Amyloid A, amyloid-β, fatty acids, fibrinogen, fibronectin, heat shock protein 60, heparan sulphate, hialuronic acid, high mobility group protein B1 (HMGB1), low density lipoprotein, α2-macroglobulin, α1-microglobulin, tenascin C, vitamin D binding protein

Classification of other LR4 protein partners [PMID 30365988]

Co-receptors or accessory molecules: Myeloid differentiation factor 2 (MD-2/Ly96), CD14, LPS binding protein (LBP).

Adaptor proteins: Myeloid differentiation primary response gene 88 (MyD88), MyD88 adaptor like (Mal/TIRAP), Toll/IL1R (TIR) domain-containing (TRIF/TICAM1), TRAM/TICAM2, IRAK.

Negative regulators: soluble (s) TLR4, TOLLIP, sMYD88, SOCS, PI3K, IRAK-M, A20, ST2L, SIGIRR/IL1-R8, TRAILR/CD261, TRIAD3A/RNF216, CD180/RP105.

Molecular steps in TLR ligation and activation (illustrated by LPS ligation) [PMID 30365988, 26136385, 27483231]

1. Binding of LPS to the co-receptor LPS binding protein (LBP).
2. LBP transports LPS to the CD14 co-receptor that exists in a soluble and a membrane-bound form.
3. Soluble CD14 form the $(CD14\text{-}LPS)_2$ dimer, membrane-bound CD14 forms a monomeric CD14/LPS complex.
4. Both the dimeric and monomeric CD14-LPS complexes can deliver LPS to the TLR4/MD-2 complex; MD-2 being another TLR4 accessory molecule (see above).
5. The new TLR4/MD-2/LPS complex can bind to either the MyD88 or the TRIF adaptor protein, and these events lead to downstream intracellular signaling. These two adaptor protein-including complexes are referred to as the Myddosome and the Triffosome complexes, respectively.

Downstream intracellular signaling [PMID 30365988, 26136385]

A. MyD88 dependent signaling: The activated TLR complex at the cell surface recruits MyD88 and initiates a signaling cascade finally leading to early NFκB activation.

B. TRIF-dependent signaling: This pathway is also referred to as MyD88 independent signaling. The activated TLR4/LPS complex is endocytosed, and this is followed by recruitment of TRIF. The downstream signaling leads to a type 1 interferon response and a later to an additional NFκB response.

Thus, both pathways lead to altered transcriptional regulation; TLR4 signaling can thereby increase the expression of cytokines/chemokines, metalloproteases and integrins.

Crosstalking pathways [PMID 29867550, 30365988**]**

TLR4 initiated signaling shows direct or indirect crosstalk with several other cellular responses or pathways, including:

(i) mitochondrial functions and production of reactive oxygen species; (ii) mitogen-activated protein kinases; (iii) AKT serine/threonine kinase (also known as protein kinase B alpha) and thereby indirect effects both on NFκB but also the hypoxia-inducible factor α; (iv) PI3K, a mediator upstream to Akt; (v) the arachidonic acid-prostaglandin system TNFα induced signaling effects.
